# A Rare Case Presentation of a Perforated Giant Sigmoid Diverticulum

**DOI:** 10.1155/2013/957152

**Published:** 2013-10-28

**Authors:** Jennifer C. Kam, Vikram Doraiswamy, Robert S. Spira

**Affiliations:** ^1^Division of Renal Diseases and Hypertension, The George Washington University School of Medicine, Washington, DC 20037, USA; ^2^Seton Hall University School of Health and Medical Sciences, St. Michael's Medical Center, Newark, NJ 07102, USA

## Abstract

Giant sigmoid diverticulum (GSD) is a rare complication of diverticulosis. These lesions arise from herniations of the mucosa through the muscle wall which progressively enlarge with colonic gas to become large air-filled cysts evident on plain X-ray and CT scans. We present a rare case of a 72-year-old female presenting with abdominal distention, abdominal tenderness, and fever who developed a type 1 giant sigmoid diverticulum (pseudodiverticulum) that subsequently formed an intra-abdominal abscess and an accompanying type 2 diverticulum as well. The patient was treated with surgical resection of the diverticulum with a primary anastomosis and abscess drainage. The patient's postoperative course was uneventful. This case helps to support the need for the consideration of GSD in patients aged 60 and older with a history of diverticulosis and presenting with abdominal discomfort and distension.

## 1. Introduction

Diverticulosis of the colon is a common clinical entity affecting 35% of individuals over the age of 65 in the western world [1, 2]. Diverticula are sac-like herniations of the mucosa through the muscle wall. The disease is usually limited to the sigmoid colon, with diverticulum typically less than 1 to 2 cm in diameter in size [2]. Giant diverticulum of the colon (GCD) is a rare condition that occurs in the sigmoid colon in more than 90% of cases [3–5]. Sigmoid diverticula infrequently enlarge to such a degree that they are termed “giant sigmoid diverticula” [2]. A giant diverticulum is defined as an air-filled cystic diverticulum larger than 4 cm in maximum diameter [1, 6]. The etiology of giant sigmoid diverticulum (GSD) is not clearly understood and can present with a variety of signs and symptoms which can include an incidental finding in an asymptomatic patient, a presentation of abdominal pain or an abdominal mass, or even an acute abdomen secondary to perforation [3, 7]. The giant diverticulum is progressively inflated by colonic gas via a hypothesized ball-valve type mechanism [1, 7–9]. The condition has also been termed giant colonic diverticulum, giant gas cyst, giant air cyst, and giant cyst [10]. We present a case of a giant sigmoid diverticulum that presented as a pseudodiverticulum with an accompanying infectious diverticulum. A brief review of giant sigmoid diverticulum is also included.

## 2. Case Presentation

**Figure 1:**
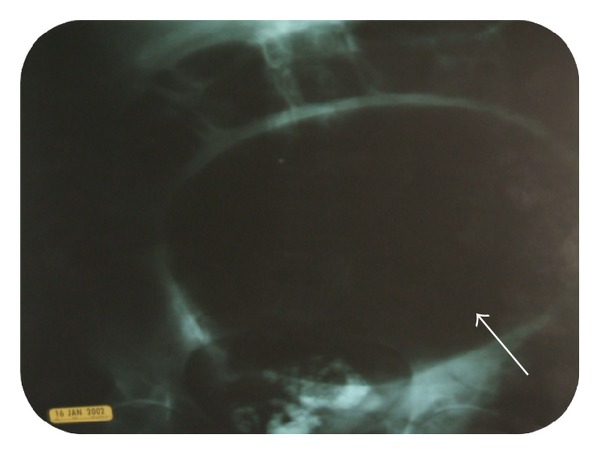
Abdominal X-ray showing air fluid level with a dilated loop of sigmoid colon.

**Figure 2:**
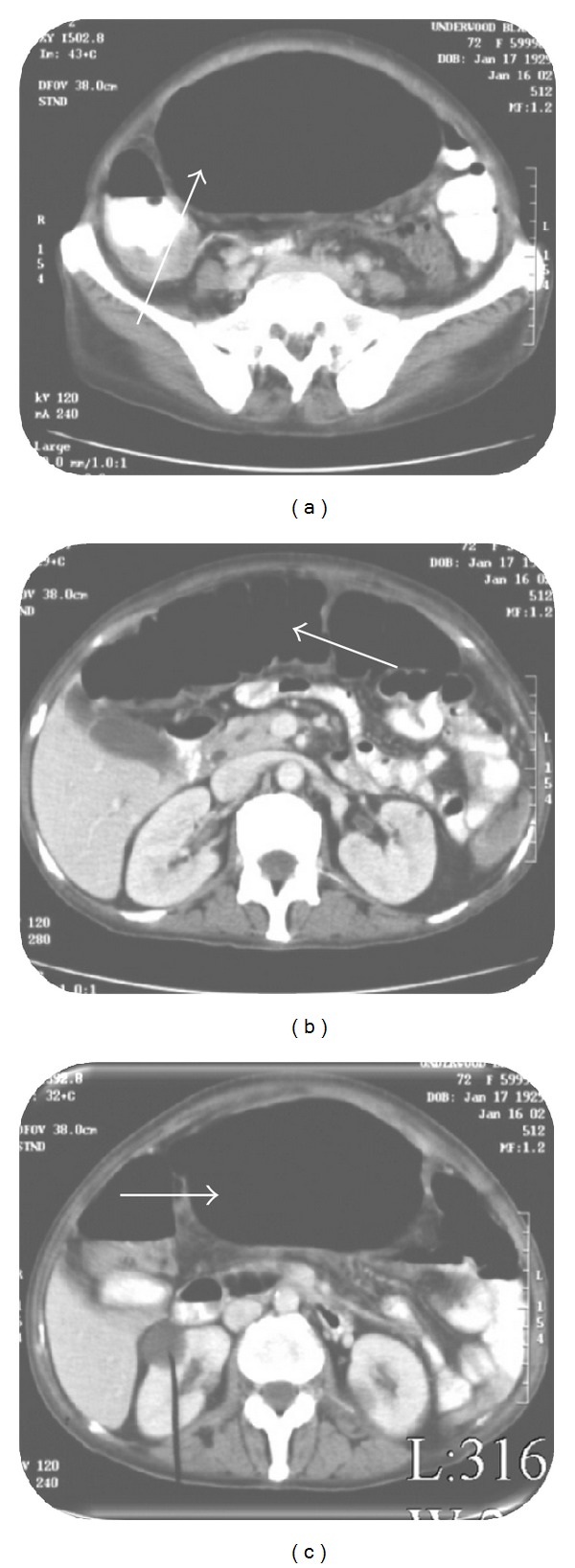
((a), (b), and (c)) Abdominal CT showing air fluid level with a dilated loop of sigmoid colon.

**Figure 3:**
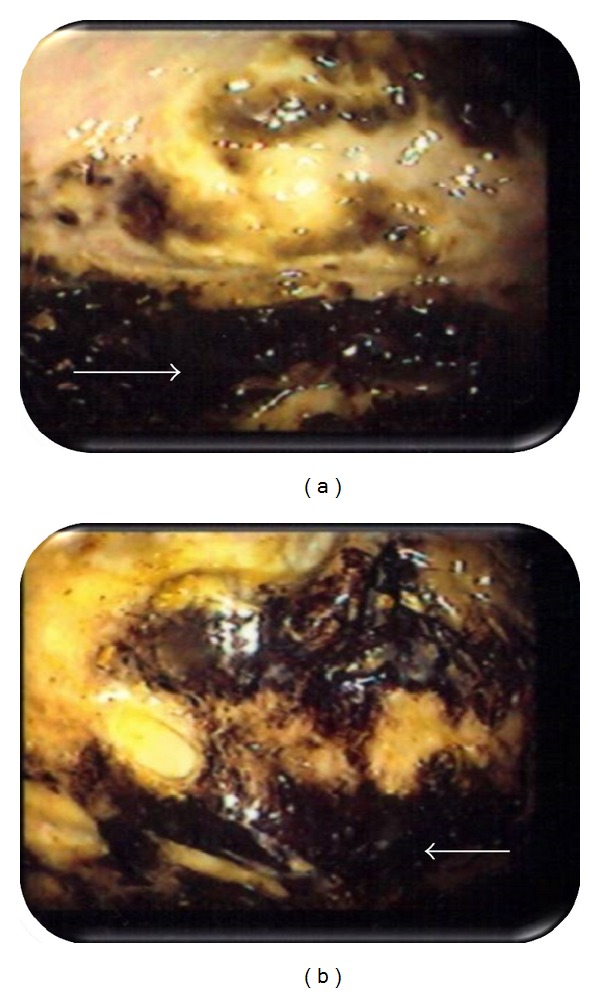
((a), (b)) Colonoscopy showing a large filled cavity lined by blackish appearing serosa (arrows).

A 72-year-old female with hypertension presented with complaints of abdominal distention, fever, decreased appetite, fatigue, and weight loss for 2 months. The patient denied any abdominal pain, cramping, vomiting, constipation, or dysphagia. On physical exam, the patient's abdomen was distended, soft, and diffusely tender with presence of normal bowel sounds. Laboratory results revealed a low hemoglobin value (7.7 g%) with a normal mean corpuscular volume, white blood cell count of 38,200 cells/mL with 21% bands, The patient was started on normal liver function, amylase, and lipase. Patient was started on ciprofloxacin, flagyl, and intravenous fluids. Plain X-ray film of the thorax was normal. A plain abdominal X-ray showed a complete filling of the upper abdomen by a giant radiolucent mass with an air-fluid level (Figure 1). Computed tomography (CT) scan of the abdomen confirmed a large, 10 cm dilated air-containing cavity with air-fluid level but without filling of the cavity with orally or intravenously given contrast medium (Figures 2(a), 2(b), and 2(c)). The patient was presumed to have volvulus, and a colonoscopy was performed. The scope was advanced into a narrowed area in the rectosigmoid region, and it entered into a large air-filled cavity lined by blackish appearing serosa (Figures 3(a) and 3(b)). The cavity lacked the lining of normal colonic mucosa. Upon continuous suctioning of air in the cavity, it completely collapsed resolving the abdominal distention. The patient was referred to surgery for suspected sigmoid pseudodiverticulum and to rule out colonic perforation. Exploratory laparotomy revealed a fresh area of perforation in the wall of sigmoid diverticulum along with an adjacent intra-abdominal abscess. Laparoscopic surgery was performed with drainage and debridement of the abscess with saline and suction. The diverticulum was successfully excised with a left hemicolectomy with primary end-to-end anastomosis, and placement of rectal Hartman's pouch with a colostomy was also performed. The histopathology of the resected specimen revealed giant sigmoid pseudodiverticulum with focal acute and chronic inflammation of serosa and subserosal tissue with neighboring type 2 inflammatory diverticula. The diverticulum was found on the antimesenteric side of the resected colon. No evidence of malignancy was identified in the excised specimen. Postoperative hospital course and recovery were uneventful, and the patient was discharged home without any complications.

## 3. Discussion

### 3.1. History

In 1943, Bonvin and Bonte reported the first case of a solitary air cyst of the peritoneal cavity attached to the sigmoid colon, subsequently called a giant diverticulum of the colon [11]. Hughes and Greene in 1953 reported the first radiological diagnosis and the first case in the English language literature and described it as a case of “solitary air cyst” arising from the antimesenteric border of the sigmoid colon. Since that time, case reports have appeared in the literature describing this condition with various names such as “giant gas cyst,” “giant sigmoid diverticulum,” “giant colonic diverticulum,” or “intestinal gas cyst” [1, 2, 5, 6, 9, 13, 14, 17]. 

### 3.2. Epidemiology

All patients with giant sigmoid diverticula present a common clinical profile. They are generally elderly, with an age range of 40 to 90 years and most occurring after the age of 60. The few reported cases of a true colonic diverticulum occurred at a slightly younger age suggesting a possible congenital component [5, 12]. Sex distribution is equal [1, 2, 7, 8, 10, 12–15]. Ninety-five percent of giant colonic diverticula are found on the antimesenteric side of the sigmoid colon [16], and in only a few cases of a true giant diverticulum was the diverticulum found on the mesenteric side of the bowel wall [5, 16]. Most giant colonic diverticulum occurs in the sigmoid colon, but several cases have been reported in the ascending, transverse, or descending colon [4, 12]. GCD size has been most frequently reported in the range of 4–9 cm and a few rarely above 25 cm [7].

### 3.3. Definition

A giant colonic diverticulum (GCD) is defined as a colonic diverticulum measuring 4 cm or larger, with a few described spanning more than 25 cm [3, 7, 10]. The size of a giant colonic diverticulum may vary over time and intermittently be palpable and hence is known to be occasionally referred to as a “phantom tumor” [10]. The size of a giant diverticulum waxes and wanes increases by straining of stools and gradually increases over years [1]. The adjacent colon is often irritable and edematous and may be compressed by the GCD [8]. The segment of colon from which the giant air cyst arises is always deformed by diverticular disease and may be simultaneously compressed by the cyst [16].

McNutt et al. divide giant diverticula into 3 types based on their pathology [2, 5, 7, 12]. 

Type 1 (22%) (pseudodiverticulum) is a type of preexisting pulsion diverticulum that arises gradually, without perforation, and its wall consists of chronic granulation and fibrous tissue with chronic inflammatory cells and remnants of muscularis mucosa [2, 5]. The true muscularis ends at the colonic border of the diverticulum [12].

Type 2 (66%) (inflammatory diverticulum) is secondary to a local perforation of the mucosa and submucosa which leads to a walled-off abscess cavity that communicates with the bowel lumen and acts as a one-way valve that allows the diverticulum to enlarge. It is lined by fibrous scar tissue without any normal intestinal layer [2, 5, 7, 12].

Type III (12%) (true diverticulum) contains all layers of normal bowel wall and is in continuity with the gut lumen, likely representing a communicating bowel duplication cyst [2, 5, 12, 15]. In rare cases, the wall may contain other substances or disease, including amyloid [17], lymphoma of mucosa-associated lymphoid tissue (MALT) [8], and urothelium [4, 15].

### 3.4. Pathogenesis

Two principal theories have been proposed for the pathogenesis of giant diverticula. A ball-valve mechanism has been suggested by Nano et al. as a cause of a gradual increase in the size of a colonic diverticulum until it transforms into GCD [12]. A pseudodiverticulum of mucosa and submucosa forms through muscularis whose communication with the bowel lumen becomes so narrowed by inflammation that a ball-valve/flap-valve mechanism is created whereby gas can enter the serosa-lined cyst as intraluminal pressure increases but cannot exit the diverticulum (a one-way communicating stalk) [1, 7–9]. Narrowing of the diverticulum neck from inflammation creates a flap-valve mechanism that traps gas in the diverticular cavity as intraluminal pressure in the bowel increases during defecation [8, 10, 12, 17]. Trapped air is vented intermittently and along with differences in colonic pressures leads to fluctuations in the GCD size [7, 12]. These changes in size of the sigmoid diverticulum have been evident in radiologic examination [5, 12, 18]. A case reported by Frankenfeld described a GDC in which the cyst waxed and waned is sized and dramatically recurred as the patient strained during bowel movements, which suggests the demonstration of a ball-valve type mechanism [9].

Another theory suggests that although the ball-valve mechanism may exist, enlargement of the diverticulum occurs in part due to gas-forming organisms located within the cyst [8]. First, the neck or stalk of the diverticulum becomes obliterated by chronic inflammation, and then gas is produced from the organisms located within the cyst, which progressively distends and enlarges the diverticulum [8, 9, 17]. The more accepted theory has been the “ball-valve theory” for several reasons. Anatomic communication is demonstrable in over two-thirds of the cases, making it difficult to believe that gas formed by microorganisms in the cyst would not vent into the lumen. Even though there has been two occasions where organisms have been cultured from the fluid within a diverticulum [8, 9], sterile cultures have been more predominantly reported [3, 8].

### 3.5. Symptoms

The clinical presentation of GCD can be variable. GCD can present as an incidental finding in an asymptomatic patient, but it can also present in patients who have clinical features suggestive of acute diverticulitis (nausea, fever, and left lower quadrant peritoneal irritation) or with more indolent features. The most consistent physical findings of giant colonic diverticula are abdominal tenderness (87%) [6] or an abdominal mass (71%) [14]. More than 80 percent of the cases are associated with a palpable mass that may or may not be tender [14]. Other frequent symptoms include nausea, vomiting, fever, constipation, diarrhea, melena, and abdominal bloating [1, 2, 4–8, 10, 12, 13, 18]. 

### 3.6. Complications

Complications of GSD occur in 15–35% of cases such as perforation, intra-abdominal abscess formation, diverticulitis, volvulus, small bowel obstruction (secondary to adhesion to the large bowel), adhesion with the bladder, and lower gastrointestinal bleeding [2, 4, 7, 8, 12, 15, 19]. The two most common complications of GSD are perforation and abscess formation [7, 12, 15]. GSD carries a mortality risk of approximately 5% [10] with a 2% risk of carcinoma (adenocarcinoma) developing inside the diverticulum [5, 15]. 

### 3.7. Diagnosis

The investigations of choice for diagnosing GCD include a plain abdominal X-ray and an abdominal CT scan; both can accurately demonstrate the classical “balloon sign” of GCD [7]. Plain abdominal X-ray films usually reveal a solitary, anteriorly placed, gas-filled cyst, varying in size from 6 to 29 cm in diameter. On the abdominal radiograph a large, smoothly marginated, round or oval, homogenous radiolucency is typically seen. Occasionally, the wall may show evidence of calcification, probably due to chronic inflammatory changes [1, 8, 10, 13]. GCDs are usually seen in the lower or midabdomen, although the position may change on subsequent radiographs [8]. An air-fluid level within the giant sigmoid diverticulum can be found in 25% of cases [1, 8, 10, 13].

Barium enema can confirm the diagnosis when there is a communication between the diverticulum and colon [1, 3, 7]. Communication between the diverticulum and sigmoid colon is demonstrated in approximately 25% of cases [3]. Barium enema is the most commonly used investigation; however, several cases of perforation have occurred within 24 h of the study, leading to the suspicion that barium enemas may precipitate perforation [2, 3, 10, 16]. 

Computed tomography is useful when barium enema fails to make the diagnosis. However, given several reported cases of perforation precipitated by barium enemas, CT scans have been shown to be adequate for the diagnosis of suspected GSD [10]. CT scanning will show a thick-walled, air-filled cavity in close apposition to the adjacent sigmoid colon. The diverticulum appears as a cavity filled with gas, fluid, or stool, with a thin regular wall and no contrast enhancement except in the presence of inflammation. The wall may contain calcifications from chronic inflammation. A thickened wall is associated with acute inflammation, correlating with diverticulitis [1, 9, 10, 15]. Sigmoidoscopy, when performed, has been uniformly noncontributory [9].

The differential diagnosis for giant colonic diverticulum includes volvulus (if associated with signs of intestinal obstruction), bowel duplication, Meckel and duodenal diverticula, infected pancreatic pseudocyst, emphysematous cholecystitis, emphysematous cystitis, vesicoenteric fistula, and intra-abdominal abscess [1, 12, 15]. Intestinal duplication cysts occur in relation to the mesenteric side of the bowel and commonly present in childhood and/or younger patients, suggesting a congenital component. These cysts usually do not communicate with the gut lumen and are rarely located in the sigmoid colon [1, 3, 8]. In contrast with a giant sigmoid diverticulum, the wall of a duplication cyst contains smooth muscle bundles. Pneumatosis cystoides intestinalis typically appears as multiple small radiolucencies in the small bowel or colon wall rather than a single large radiolucency [3, 8].

### 3.8. Treatment

Resection of the diverticulum and adjacent sigmoid colon is the preferred treatment in uncomplicated GCD [1, 3, 5, 7–10, 15]. For patients presenting with complications, such as a coexisting carcinoma, resection with Hartmann's procedure (a two-stage bowel resection with colostomy and mucous fistula or Hartmann pouch) is necessary [1, 3, 5, 7, 10, 15, 19]. In cases where there is free perforation or localized abscess formation, percutaneous drainage is recommended [1, 3, 7, 10, 15]. Conservative management without surgery should be reserved only for high-risk patients who are unable to tolerate surgery or who are unwilling to have surgery [1, 10].

## 4. Conclusion

Giant sigmoid diverticulum is a rare manifestation of colonic diverticulosis. We present a rare case of a patient who developed a type 1 giant sigmoid diverticulum (pseudodiverticulum), which further developed an accompanying type 2 diverticulum (inflammatory). An intra-abdominal abscess formed as the sequelae of the inflammatory diverticula. 

Perforation is a known complication of GCD, as evidenced in the patient described above. It is likely that more than one pseudodiverticulum was present, one found incidentally and the other forming an abscess leading to the patients physical findings of abdominal distention.

This case helps to support the need for the consideration of GCD in patients aged 60 and older with a history of diverticulosis and presenting with abdominal pain and distension. This case is unique in that it is a rare presentation of a GSD that presented as a type 1 diverticulum with accompanying type 2 diverticula. A left hemicolectomy, rectal Hartman's pouch, and colostomy were performed, and the patient was subsequently discharged without complications.
